# Genetic status of *KRAS* modulates the role of Neuropilin-1 in tumorigenesis

**DOI:** 10.1038/s41598-017-12992-2

**Published:** 2017-10-10

**Authors:** Sneha Vivekanandhan, Lijuan Yang, Ying Cao, Engfeng Wang, Shamit K. Dutta, Anil K. Sharma, Debabrata Mukhopadhyay

**Affiliations:** 1 Department of Biochemistry and Molecular Biology, Mayo Clinic College of Medicine and Sciences, Rochester, MN USA; 2 Department of Biochemistry and Molecular Biology, Mayo Clinic College of Medicine and Sciences, Jacksonville, FL USA

## Abstract

Neuropilin-1 (NRP1), a non–tyrosine kinase receptor, is overexpressed in many cancers including pancreatic and lung cancers. Inhibition of NRP1 expression, however, has differing pro-tumor *vs*. anti-tumor effects, depending on the cancer types. To understand the differential role of NRP1 in tumorigenesis process, we utilized cells from two different cancer types, pancreatic and lung, each containing either wild type KRAS (*KRAS* ^wt^) or mutant KRAS (*KRAS* ^mt^). Inhibition of NRP1 expression by shRNA in both pancreatic and lung cancer cells containing dominant active *KRAS* ^mt^ caused increased cell viability and tumor growth. On the contrary, inhibition of NRP1, in the tumor cells containing *KRAS* ^wt^ showed decreased tumor growth. Importantly, concurrent inhibition of *KRAS* ^mt^ and NRP1 in the tumor cells reverses the increased viability and leads to tumor inhibition. We found that NRP1 shRNA expressing *KRAS* ^mt^ tumor cells caused increased cell viability by decreasing SMAD2 phosphorylation. Our findings demonstrate that the effects of NRP1 knockdown in cancer cells are dependent on the genetic status of *KRAS*.

## Introduction

Neuropilin-1 (NRP1) is a non–tyrosine kinase receptor involved in neuronal guidance and angiogenesis^1,2^. Recent studies have shown that NRP1 is multifunctional and capable of binding multiple growth factors and mediators, including integrins, fibroblast growth factors, transforming growth factor β-1 (TGFβ1) and its receptors, hepatocyte growth factor and its receptor c-Met, and platelet-derived growth factor and its receptors^3,4^. These interactions link NRP1 to multiple pathways including tumorigenesis and angiogenesis. A growing collection of literature have shown that increased expression of NRP1 correlates with tumor progression and poor prognosis in hematologic malignancies, such as acute leukemia^5–7^ and various solid tumors, including pancreatic cancer^8^ and lung cancer^9,10^. In several cancers like lung cancer^11^ and renal cancer^12^ blocking of NRP1 expression has been shown to suppress tumor growth. Conversely, NRP1 overexpression has been shown to promote tumor progression in certain cancers, glioma^13^, prostate cancer^14,15^, and colon cancer^16^. Thereby, it was assumed that NRP1 might constitute a promising target for cancer therapy.

Interestingly, differing from the established notion that NRP1 promotes tumor growth, Gray *et al*.^17^ showed that overexpression of NRP1 in pancreatic cancer could decrease tumor incidence and inhibit tumor growth, whereas reduced expression of NRP1 by small interfering RNA enhanced tumor growth. Therefore, the experimental data on the role of NRP1 in cancer are conflicting, and the molecular mechanisms of differential role of NRP1 in tumorigenesis remain unknown.

Here, we would like to elucidate the mechanism through which NRP1 plays opposing roles in oncogenesis and tumor progression. We demonstrated a novel link between NRP1 and the genetic status of *KRAS* in tumorigenesis. Our results showed that NRP1 has different effects in pancreatic and lung cancers depending on their *KRAS* genotype. We further established the role of the TGFβ/SMAD pathway in NRP1/KRAS-interdependent signaling. In cancer cells harboring *KRAS* ^mt^, NRP1 down regulation promotes tumorigenesis through the suppression of SMAD2 pathway. These novel findings provide an opportunity to tailor precise, individualized therapies for effective cancer treatment.

## Results

### NRP1 has opposing effects on *in vitro* cell viability of different cancer cell lines

**Figure 1:**
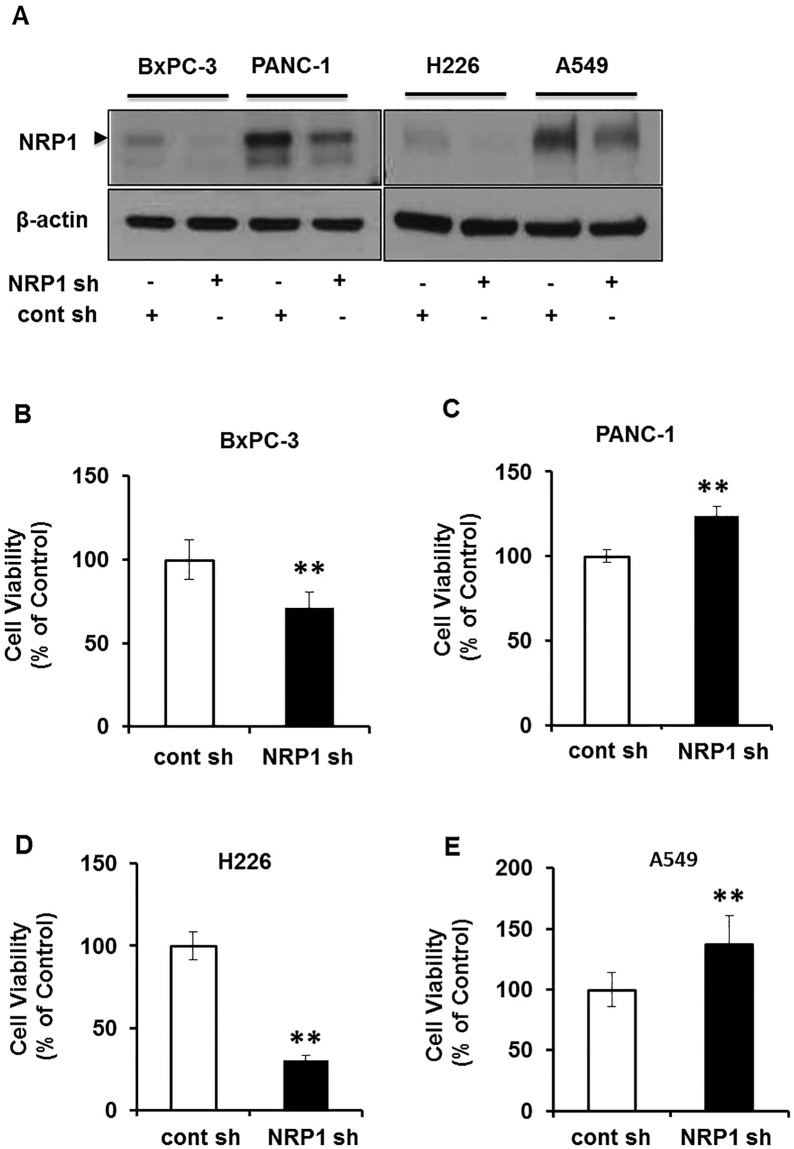
Oncogenic vs wild-type *KRAS* influences the role of NRP1 in tumorigenesis in pancreatic and lung cancers. (**A**) Western blot analysis of NRP1 knockdown levels in PDAC and NSCLC cells. (**B**–**E**) Cell viability for different PDAC cells BxPC-3 (**B**) and PANC-1 (**C**) and NSCLC cells H226 (**D**) and A549 (**E**) with reduced NRP1 expression grown in 3D culture for 72 hours. Data are plotted as percentage of control cells (transfected control shRNA -cont sh). All data are presented as the mean ± SD of 3 independent experiments. *P < 0.05 vs control; **P < 0.01 vs control.

To understand the differential role of NRP1 in different cancers, we utilized short-hairpin RNA (shRNA)-mediated NRP1 knockdown in human pancreatic cancer (PDAC) and non–small cell lung cancer (NSCLC) cell lines. NRP1 knockdown was confirmed by Western blot analysis (Fig. 1A, Supplemental Figure 8A). We examined the cell viability of the tumor cells using 3-dimensional (3D) cell culture systems. Cell viability was confirmed using a luminescent cell viability assay. After NRP1 knockdown, viability was increased in PDAC and NSCLC cancer cell lines with *KRAS* ^mt^, PANC-1 and A549 respectively and was decreased in cells with *KRAS* ^wt^, BxPC-3 and H226 respectively (Fig. 1B–E). A similar trend was observed in cell viability assays in 2D cell cultures (Supplemental Fig. 1A–D). We also noticed a similar trend in another *KRAS* ^mt^ PDAC cell line, AsPC-1 (Supplemental Fig. 1E and F). We then examined if the increased viability was due to increased proliferation or decreased cell death. We found that in PANC-1 and A549 cells there was increased phosphorylation of extracellular signal-regulated kinase ERK 44/42 at the protein levels when NRP1 expression was reduced using shRNA (Supplemental Fig. 1G,H). These data suggest that in cells with *KRAS* ^mt^, downregulation of NRP1 leads to increased viability likely through increased cell proliferation. Taken together, these results indicate that NRP1 has distinct viability effects between different PDAC and NSCLC cancer cell lines.

### NRP1 has opposing effects on the tumor growth based on KRAS genetic status *in vivo*

**Figure 2:**
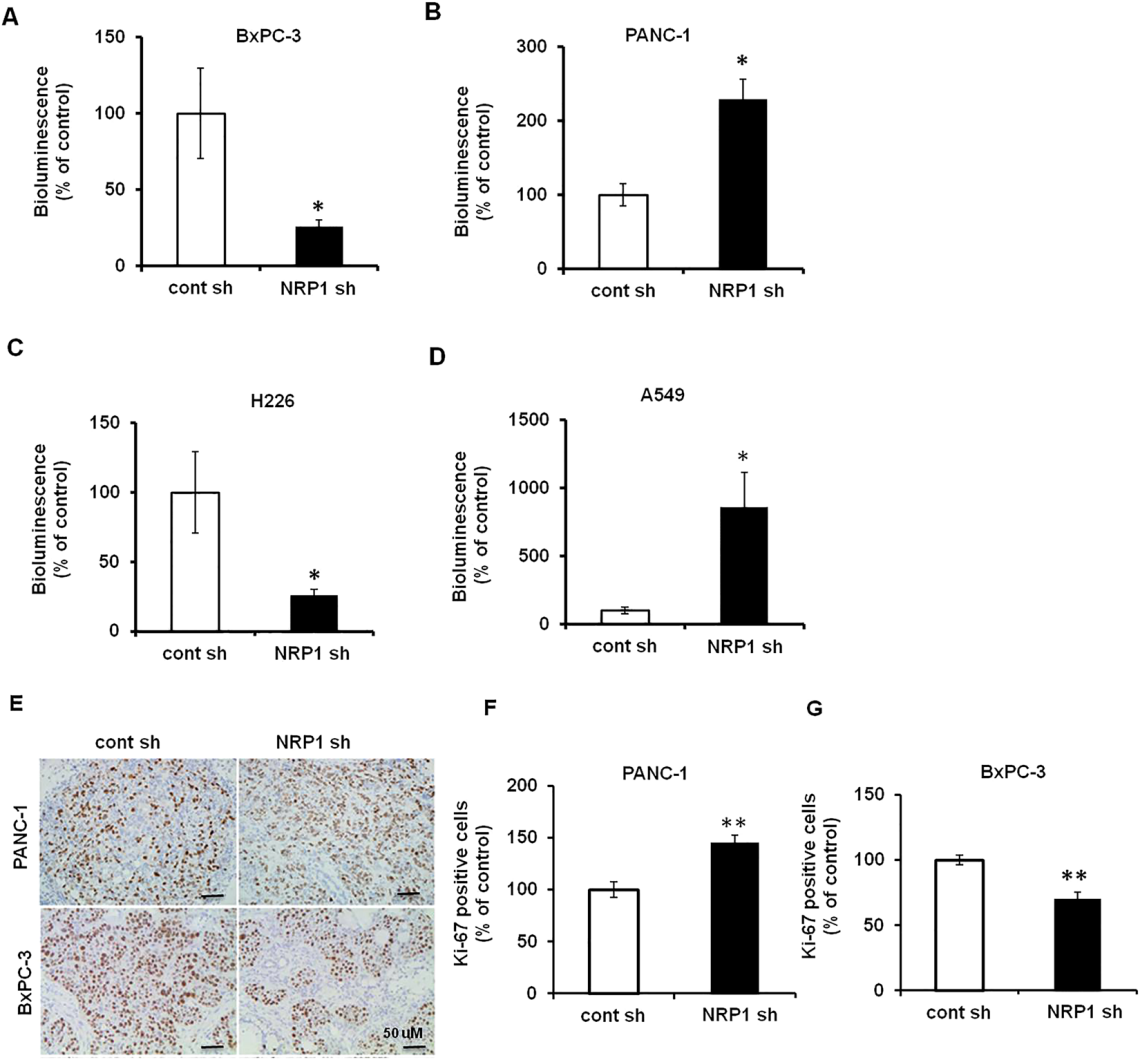
Role of NRP1 in tumorigenesis is different in wild-type vs mutant *KRAS* tumor models. SCID mice were implanted with different PDAC (orthotopic model) and NSCLC (tumor xenograft model) cell lines with or without NRP1 knockdown. (**A**–**D**) Quantification of the mean ± SD bioluminescent signals (n = 5 for each group). (**E**) Immunohistochemical staining for Ki-67 in different pancreatic cancer cell lines. (**F**,**G**) Quantification of digital images for Ki-67 staining at ×20 magnification. *P < 0.05 vs control; **P < 0.01 vs control.

To further validate this varying role of NRP1 in tumorigenesis in different cancers through *in vivo* studies, we utilized orthotopic human PDAC and NSCLC tumor xenografts in immunocompromised SCID mice. Tumor volume was monitored by bioluminescence imaging and quantified with imaging software. NRP1 downregulation in the PDAC cell line PANC-1 and NSCLC cell line A549 (*KRAS* ^mt^) resulted in increased tumor growth, whereas NRP1-deficient PDAC BxPC-3 cells and NSCLC H226 cells (*KRAS* ^wt^) exhibited decreased tumor growth as compared to that of parental cells (Fig. 2A–D; Supplemental Fig. 2A).

Tumor tissues were further analyzed by immunohistochemistry for Ki-67, which serves as a marker for proliferation. Tumors formed in mice harboring NRP1 shRNA - PANC-1 and A549 cells had significantly more Ki-67–positive cells than controls, whereas tumors derived from BxPC-3 and H226 cells had significantly lower nuclear expression of Ki-67 than controls (Fig. 2E–G; Supplemental Fig. 2B–D). Thus, our *in vivo* data were consistent with our *in vitro* data proposing that NRP1 deficiency in *KRAS* ^mt^ cells was pro-tumorigenic but seems to inhibit tumor growth in *KRAS* ^wt^ cells.

### The role of NRP1 in tumorigenesis is oncogenic *KRAS* dependent

Our results corroborate the published literature suggesting that NRP1 exhibits contrasting effects on tumorigenesis. Although the associated molecular mechanism is poorly understood, NRP1 is known to require an effector molecule because it lacks intrinsic kinase activity. We previously reported that wild-type KRAS is one of the downstream effector molecules of NRP1^18^, which could mediate its opposing functions in tumor growth and progression. Since *KRAS* mutation is among the most common oncogenic mutations associated with tumor growth, we hypothesized that *KRAS* mutation status may influence the role of NRP1 in tumorigenesis. Correspondingly, our data indicate that NRP1 knockdown in *KRAS* ^mt^ cancer cells (PANC-1, A549) promotes tumorigenesis, whereas NRP1 down regulation in *KRAS* ^wt^ cells (BxPC-3, H226) inhibits tumorigenesis (Supplemental Table 1). Furthermore, a review of relevant published reports detailing the effects of NRP1 in various tumors supports the premise that down regulation of NRP1 promotes tumorigenesis in the presence of *KRAS* ^mt^ and suppresses tumor formation with *KRAS* ^wt^. These findings support our hypothesis that the *KRAS* mutation status of cancer cells influences whether NRP1 promotes or suppresses tumor growth in that particular type of cancer cells (Supplemental Table 2)^12,13,15–17,19–22^.

**Figure 3:**
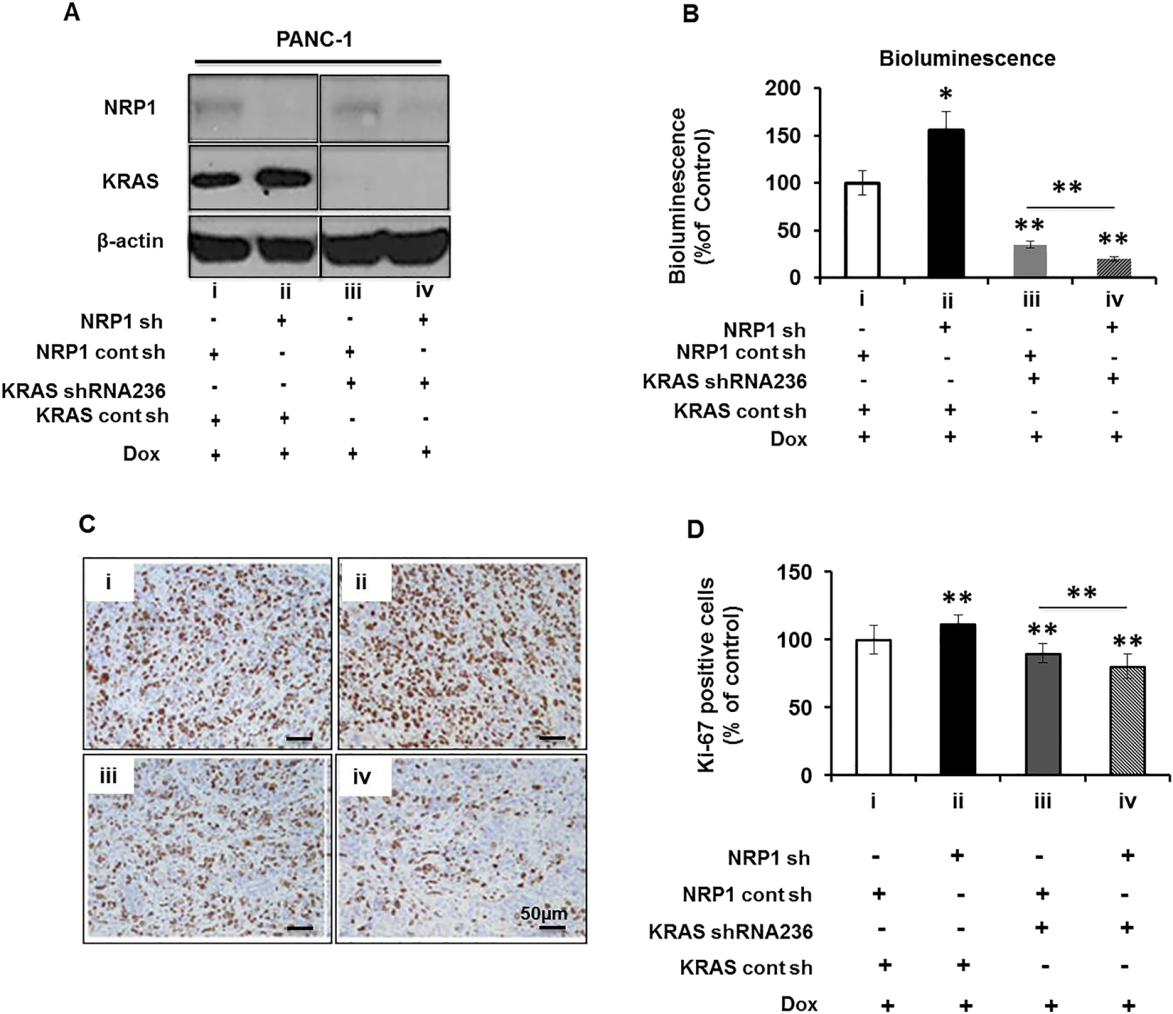
Role of NRP1 in tumorigenesis is oncogenic *KRAS* dependent. (**A**) Western blot analysis showing NRP1 and KRAS levels in doxycycline (Dox)-inducible PANC-1 cells. (**B**–**D**) Four groups of SCID mice (n = 8 each) had (i) orthotopic implantation of PANC-1 cells transfected with NRP1 control and KRAS control shRNA or (ii) NRP1 shRNA and KRAS control shRNA; (iii) NRP1 control shRNA and KRAS 236 shRNA (loss of KRAS expression) or (iv) NRP1 shRNA and KRAS 236 shRNA (loss of KRAS and NRP1 expression). All groups received 0.1 μg/mL Dox treatment throughout the entire 8-week study period. (**B**) Quantification of bioluminescence signals before sacrifice. (**C**) Ki-67 immunohistochemical staining in each group. (**D**) Quantification of digital images for Ki-67 staining at ×20 magnification. *P < 0.05 vs control; **P < 0.01 vs control. ^#^ *P* < 0.05; ^##^ *P* < 0.01 between groups (iii) and (iv).

To confirm the involvement of KRAS in modulating the role of NRP1, we expressed doxycycline-inducible KRAS shRNA in PANC-1 cells in the presence and absence of shRNA-mediated NRP1 knockdown (Fig. 3A; quantifications Supplemental Figure 8B). These cells were orthotopically injected into SCID mice to generate tumors. Tumor volume was monitored by bioluminescence imaging and quantified using imaging software (Supplemental Fig. 3). The reduced expression of NRP1 alone in the *KRAS* ^mt^ PANC-1 cells resulted in enhanced luciferase expression, but luciferase expression was significantly lower in mice with tumors lacking both KRAS and NRP1 compared to that of KRAS knockdown alone (Fig. 3B). The same trend was found in tumor volumes (Supplemental Fig. 3B). These results confirm previous reports that KRAS is important for proliferation^23,24^.

Assessment of proliferation by immunohistochemical staining for Ki-67 (Fig. 3C and D) showed that tumors had more Ki-67–positive cells after NRP1 knockdown compared with the control group. Conversely, tumors lacking KRAS showed fewer Ki-67–positive cells, and tumors deficient in both KRAS and NRP1 showed the least staining for Ki-67. These data indicate the involvement of KRAS in NRP1-mediated tumorigenicity.

To investigate for the effects of NRP1 on mutant and wild-type KRAS, we assessed KRAS protein levels in *KRAS* ^mt^ and *KRAS* ^wt^ cells after NRP1 knockdown using Western blot. KRAS levels sometimes were upregulated in *KRAS* ^mt^ cells after NRP1 knockdown compared with controls and sometimes were not (Supplemental Fig. 4A). In co-immunoprecipitation assays, NRP1 and KRAS were present in the same immunocomplex in all cell lines (PANC-1, BxPC-3, A549, and H226) (Supplemental Fig. 4B and C). Our findings suggest that NRP1 may modulate tumorigenesis through a different signaling pathway.

### TGFβ influences NRP1 levels in cancer cells

**Figure 4:**
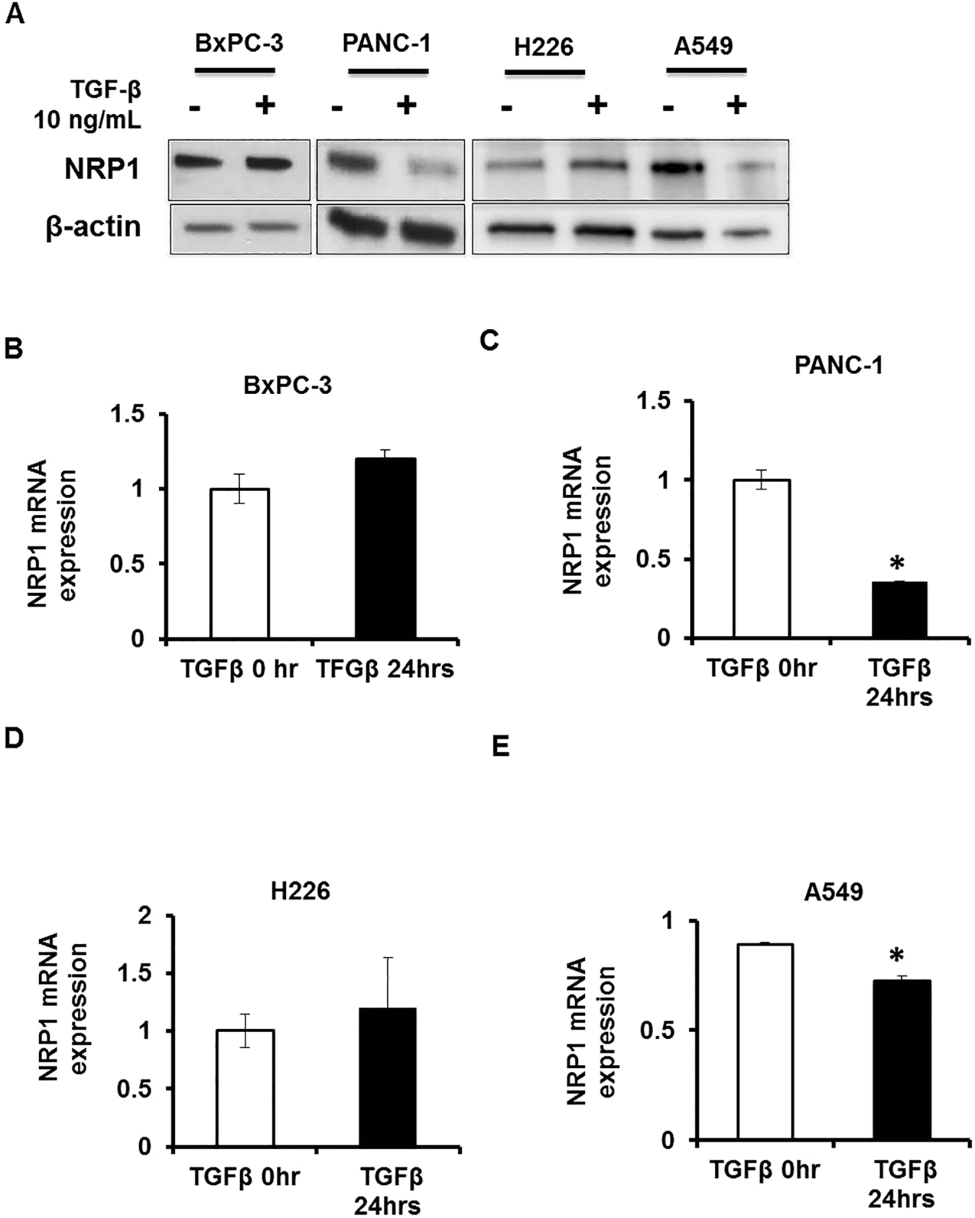
TGFβ regulates NRP1 expression levels in cancer cells based on oncogenic *KRAS* status. (**A**) Western blots showing the levels of NRP1 after 24 hours of TGFβ induction. (**B**–**E**) mRNA expression levels of NRP1 in PDAC and NSCLC cells after 24 hours of TGFβ induction. *P < 0.05 vs control **P < 0.01 vs control.

Next, we studied the biological significance of our findings. For our *in vitro* and *in vivo* studies, we used shRNA to knock down NRP1 in the cell lines. We were interested in exploring how NRP1 expression was being downregulated in the actual setting. Most cancer cells and stellate cells secrete various cytokines, such as TGFβ^25^, that influence both cancer cells and stromal cells. Our group previously demonstrated that NRP1 mediates divergent receptor-regulated SMAD (RSMAD) signaling upon TGFβ stimulation to modulate myofibroblast phenotype^26^. Based on this finding, we investigated whether TGFβ affects NRP1 expression or function by stimulating *KRAS* ^mt^ (PANC-1, A549) and *KRAS* ^wt^ (BxPC-3, H226) cells with TGFβ for 24 hours. The protein expression level of NRP1 decreased after TGFβ treatment in *KRAS* ^mt^ cells (PANC-1, A549) (Fig. 4A; quantifications Supplemental Figure 8C). We next examined mRNA expression levels to investigate how NRP1 expression was being regulated. NRP1 mRNA expression was significantly decreased after TGFβ treatment in PANC-1 and A549 cells and was slightly upregulated in BxPC-3 and H226 cells (Fig. 4B–E). The decrease in NRP1 level in A549 cells was not as much as in PANC-1 cells. It is possible that in A549 cells NRP1 protein level is modulated additionally at the post-transcriptional stage. Hence, our results show that TGFβ can downregulate NRP1 expression in *KRAS* ^mt^ cells (PANC-1, A549) thereby contributing to tumor growth in our model.

Further, as we had earlier seen that in PANC-1 cells with reduced NRP1 expression there is increased phosphorylation of ERK (p44/42) we examined the effect of TGFβ treatment on the phosphorylation of ERK (p44/42). We observed that there was increased ERK (p44/42) phosphorylation in PANC-1 cells with reduced NRP1expression on TGFβ treatment (Supplemental Fig. 5). These data suggest that ERK1/2 possibly helps to promote tumorigenesis in our model.

### Oncogenic *KRAS* differentially regulates RSMAD signaling

Our earlier work showed that, in stromal fibroblast cell lines, NRP1 knockdown resulted in increased SMAD1/5 phosphorylation and decreased SMAD2/3 phosphorylation on TGFβ stimulation^26^. Given that, and our current finding that TGFβ can modulate NRP1 levels, we investigated whether NRP1 modulates tumorigenesis via SMAD signaling. From here on, our study focuses on the pancreatic cancer cell lines.

**Figure 5:**
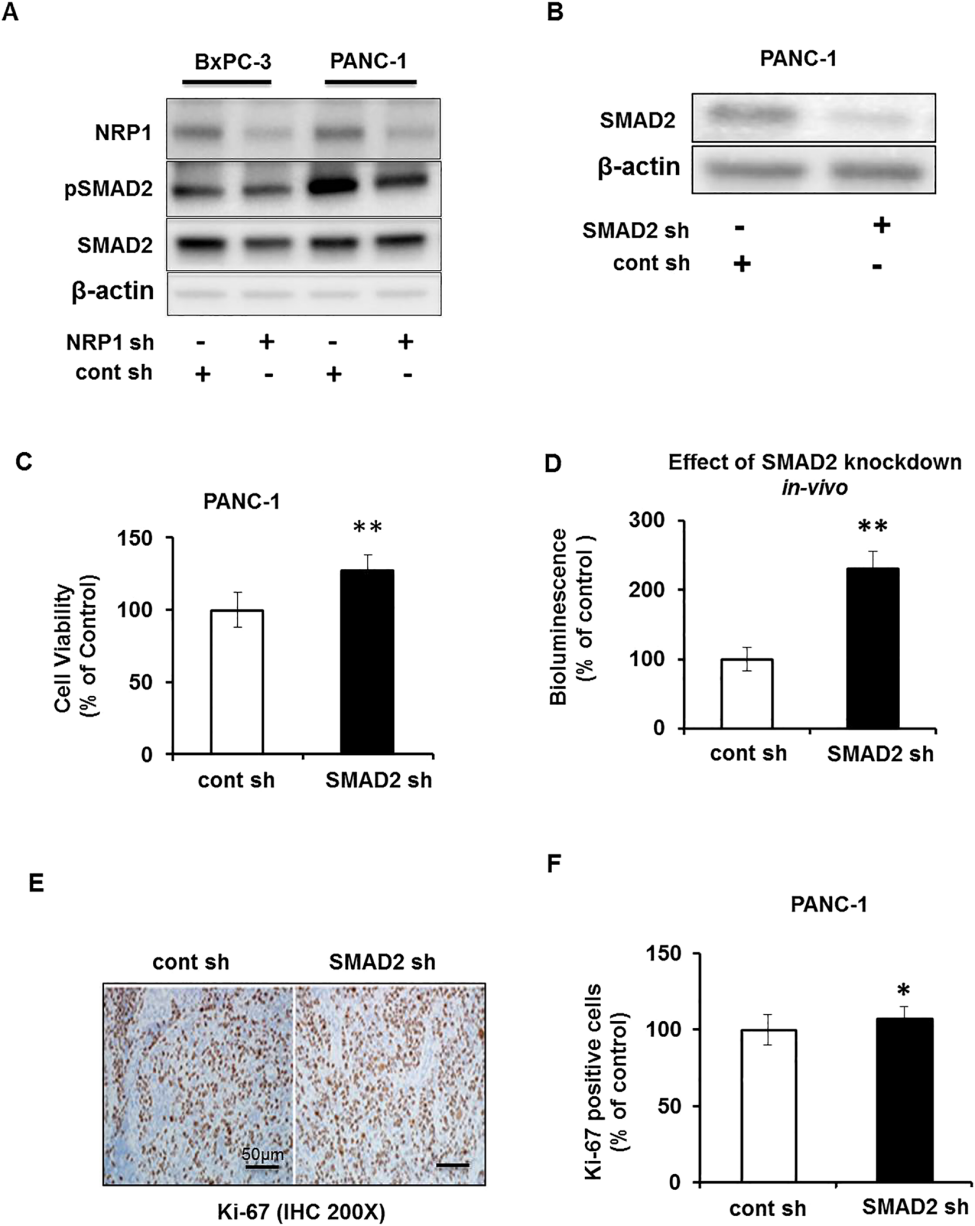
NRP1 affects tumorigenesis through divergent SMAD signaling. (**A**) Western blot showing levels of total and phosphorylated SMAD2 in pancreatic cancer cell lines with and without shRNA-mediated NRP1 knockdown. (**B**) Western blot showing the shRNA-mediated knockdown of SMAD2 in PANC-1 cells. (**C**) Cell viability for PANC-1 cell line with knockdown of SMAD2 grown in 2D culture for 72 hours. (**D**) SCID mice were orthotopically implanted with PANC-1 cells with SMAD2 knockdown. Graph shows quantification of the bioluminescent signals before the mice were sacrificed. Values represent mean ± SD of the group. (**E**) Pancreatic tissue sections from mice xenografts of PANC-1 cells with and without SMAD2, stained immunohistochemically for Ki-67. The representative image is at ×20 magnifications. (**F**) Quantification of Ki-67 staining in the mouse xenografts (n = 3 per group). Statistical analysis was determined using 1-way ANOVA. *P < 0.05 vs control; **P < 0.01 vs control.

Analysis of phosphorylation levels of SMAD2 in pancreatic cell lines deficient for NRP1 showed decreased phosphorylation in *KRAS* ^mt^ PANC-1 cells compared with control cells (Fig. 5A; quantifications Supplemental  Fig. 8D). Interestingly, in the *KRAS* ^wt^ BxPC-3 cells, there was no change in the phosphorylation of SMAD2. A similar pattern was observed in PANC-1 cells treated with siRNA against NRP1. We observed that in PANC-1 NRP1 siRNA treated cells the phosphorylation of SMAD2 was decreased as compared to the control forty-five minutes post TGFβ induction (Supplemental  Fig. 6).

To study the effect of SMAD2 on tumorigenesis, we generated SMAD2-deficient PANC-1 cells by transfecting them with a SMAD2-specific shRNA construct. The effective knockdown of SMAD2 was confirmed via Western blot analysis (Fig. 5B). The cell viability of SMAD2-deficient PANC-1 cells *in vitro* showed a significant increase as compared to that of parental lines (Fig. 5C). These SMAD2-deficient stable PANC-1 cells were injected into a murine orthotopic pancreatic cancer model. As shown in Fig. 5D, the loss of SMAD2 increased luciferase expression compared with the control group. Examination of the proliferation marker Ki-67 showed significantly increased Ki-67–positive cells in the SMAD2 knockdown group compared to that of the parental line (Fig. 5E and F). Taken together, these results suggest that SMAD2-silenced cells have a significantly more aggressive phenotype, that SMAD2 decreases cell viability in the PANC-1 cell line, and that NRP1/KRAS might modulate tumorigenesis process through SMAD2 signaling. Assessment for the potential downstream target of SMAD2 that might be involved showed that in PANC-1 cells, the expression of Serpine was upregulated at the mRNA level twenty-four hours post TGFβ treatment. However, we did not observe a change in the Serpine mRNA level in BxPC-3 after TGFβ induction (Supplemental  Fig. 7F).

## Discussion

NRP1 was originally defined as a co-receptor for many growth factors and has been associated with tumors growth and angiogenesis. The role of NRP1 in tumorigenesis is complex and context dependent. Published data indicate that upregulation of NRPs correlates with tumor progression, advanced disease stage, and poor prognosis in various cancers. However, the experimental data investigating the functional role of NRPs in cancer are inconsistent^11–17,19,20^. Several factors can account for these varying the results. It is possible that the expression of the functional receptor/ligand repertoire influences the function of NRP1 in tumorigenesis. NRP1 can mediate different functions by associating with different ligands and their signal transducers. For example, NRP1 is a co-receptor for both plexins and vascular endothelial growth factor (VEGF) receptors. Semaphorin (SEMA) 3 F and VEGF_165_ have been shown to compete for NRP receptors on the cell surface^27^. By associating with plexins, NRP1 transduces SEMA signaling, whereas it transduces VEGF signals when associated with VEGF receptors. SEMA3B and SEMA3F have been implicated in mediating tumor-suppressing effects, whereas VEGFA has been shown to promote tumor cell proliferation and survival by binding to NRP1^27–33^. Similarly, NRP1 can associate with GIPC1 and promote tumor progression through the KRAS/ERK signaling pathway^34^.

All of the initially published data examining the role of NRP1 in cancers showed that NRP1 stimulated tumor growth^12,13,15,19–22^. We have also previously shown that NRP1 promoted an undifferentiated phenotype in cancer cells^12^. Later, Gray and colleagues^17^ demonstrated that NRP1 could suppress tumor growth in the pancreatic adenocarcinoma cell line PANC-1, thereby opposing the existing notion that NRP1 promotes tumors. The relevant literature suggests that these discrepancies may be attributed to tissue and cell type differences or *in vitro* versus *in vivo* differences^17,35–37^. We carefully scrutinized these differences and found that the mutation status of *KRAS* in tumors most likely affects the role of NRP1 in tumorigenicity. In the current study, we demonstrated that the knockdown of NRP1 promotes tumor formation in *KRAS* ^mt^ cells (PANC-1, A549, and AsPC-1) but suppresses tumor growth in *KRAS* ^wt^ tumor cells (BxPC-3, H226). The knockdown of both KRAS and NRP1 in PANC-1 cell lines (*KRAS* ^mt^) inhibited tumor formation. These data suggest that the *KRAS* genotype is involved in NRP1modulated tumorigenesis. Of importance, *KRAS* is mutated in more than 90% of pancreatic cancers and 35% of lung adenocarcinomas^23,38^.

To understand the biological relevance of our studies, we investigated how NRP1 might be downregulated in actual cellular settings. Cancer cells and stellate cells are known to secrete various cytokines including TGFβ^25^. The TGFβ pathway is known to induce epithelial-mesenchymal transition, which results in an increased ability to invade and metastasize^39^. We have previously demonstrated that NRP1 regulates the myofibroblast phenotype through divergent RSMAD signaling^26^. Supporting our finding, Glinka *et al*. published NRP1 acts as a TGFβ co-receptor via SMAD2/3 signaling^40^. Furthermore, other studies have reported the interaction of RAS signaling with the TGFβ family, in which the antitumor effect of TGFβ is altered to pro-tumor signaling via SMAD proteins^41^. On the basis of these previous findings, we analyzed whether TGFβ and the canonical TGFβ pathway could be involved in *KRAS* ^mt^/NRP1-mediated tumorigenesis. In cells with *KRAS* ^mt^ (PANC-1 and A549), NRP1 expression is decreased at the protein level 24 hours after stimulation with TGFβ. The mechanism regulating NRP1 downregulation in different cell types is different. In PANC-1 cells, NRP1 is downregulated at the mRNA level, whereas in A549 cells protein stability may be affected in addition to the decrease in mRNA level.

To delineate the pathway involved in NRP1 signaling, we analyzed phosphorylation levels of SMAD2. Down regulation of NRP1 in *KRAS* ^mt^ cells decreased the phosphorylation of SMAD2, but there was no change in SMAD2 phosphorylation in *KRAS* ^wt^ cancer cells upon NRP1 knockdown. Using PANC-1 cells with confirmed SMAD2 knockdown in orthotopic mice models; we observed an increase in tumors compared with controls. This is in agreement with our *in vitro* findings that SMAD2 has an antitumor role in our model. In *KRAS* ^mt^ cells, NRP1 knockdown results in decreased SMAD2 phosphorylation and enhanced tumor growth. SMAD2 knockdown via shRNA in *KRAS* ^mt^ cells also led to increased tumor growth. *KRAS* ^wt^ BxPC-3 cells did not survive after shRNA-mediated SMAD2 knockdown.

Regarding potential downstream targets, a publication by Chow *et al*.^42^ reported that in PDAC cells, oncogenic KRAS/ERK helps in TGFβ-induced downregulation of PTEN at the mRNA level, which in turn could help promote tumor growth. ERK1/2 has been reported to promote pancreatic  cancer^43^, ovarian cancer^44^, and prostate cancer^45^. We found that phosphorylated ERK1/2 was upregulated in PANC-1 cells with reduced NRP1 24 hours after TGFβ stimulation. This result agrees with the literature suggesting that ERK1/2 promotes tumor growth and likely facilitates it in our model, as well.

Serpine is a SMAD2/TGFβ target^46,47^. It has been published that SerpinB1 can promote pancreatic β cell proliferation^48^. Our results show that Serpine is upregulated at the mRNA levels in PANC-1 cells post 24hrs of TGFβ induction. This data suggests that Serpine might likely help promote tumor growth in *KRAS* ^mt^ cells. However, more detailed studies are needed to fully understand the role of Serpine in this cross -talk.

In summary, our study provides some insight regarding the pro-tumor and anti-tumor functioning of NRP1 in different cancer types. Our results demonstrate that the genetic status of *KRAS* influences the role of NRP1 in tumorigenesis. We found that in *KRAS* ^mt^ cells, NRP1 acts as a tumor suppressor. NRP1 knockdown enhanced cell viability and tumor growth and resulted in decreased SMAD2 phosphorylation. However, in *KRAS* ^wt^ cells, NRP1 knockdown suppressed cell viability and tumor growth. Future studies are needed to fully understand the regulatory circuitry involved.

## Materials and Methods

### Cell Culture

The human pancreatic ductal adenocarcinoma cell line PANC-1 was maintained in Dulbecco’s Modified Eagle Medium plus 10% fetal bovine serum (FBS), 1% antibiotic-antimycotic (anti-anti; Gibco), and 0.02% plasmocin (Invivogen, USA). Human pancreatic adenocarcinoma cell lines AsPC-1 and BxPC-3 were maintained in RPMI 1640 medium plus 10% FBS, 1% anti-anti, and 0.02% plasmocin. Lung cancer cell lines A549 and H226 were maintained in RPMI 1640 medium plus 10% FBS, 1% anti-anti, and 0.02% plasmocin. PANC-1 KRAS-inducible cell lines were maintained in 10% tetracycline-free FBS plus 1% anti-anti and 0.02% plasmocin. All cell lines were purchased from American Type Culture Collection. Cells were serum starved for 16 h before TGFβ (Biolegend, USA) treatment. For 3D cell culture, cells were grown in medium containing carboxymethylcellulose at a ratio of 8:2 prepared as previously described^49^.

### shRNA Transfection

The plasmids for NRP1 shRNA and control shRNA were purchased from Open Biosystems, US. Dr. Francesco Hofmann^50^ (Novartis Institutes for Biomedical Research, Oncology Disease Area, Basel, Switzerland) generously provided us with the shRNA plasmids for KRAS (sh236) and control (shNT). Lentiviruses for NRP1 shRNA and control shRNA were prepared and infected into the target cells as described^51^. After infection, 2 μg/mL of puromycin was added to the medium for antibiotic selection. For the Tet-On–inducible KRAS shRNA, 0.1 μg/mL of doxycycline was used to induce shRNA expression in the stably infected cells. The sequences of the shRNAs used are listed in Supplementary Table 3.

### Cell Proliferation Assays

Cells (3,000) were seeded into 96-well plates and cultured for 72 hours in complete medium. After 72 hours, the MTS cell proliferation assay (CellTiter 96® AQueous One Solution Cell Proliferation Assay (MTS) Promega, Madison WI) was performed for 2D cell culture; the CellTiter-Glo Luminescent Cell Viability Assay (Promega, Madison WI) was used for cells grown in 3D cell culture.

### Antibodies

Western blot antibodies for β-actin, KRAS and horseradish peroxidase–conjugated secondary antibodies were purchased from Santa Cruz Biotechnology. Antibodies to NRP1, SMAD2 and p-SMAD2 were purchased from Cell Signaling Technology Inc, Danvers, MA. Ki-67 antibody used for immunohistochemistry was purchased from Santa Cruz, US.

### Preparation of Whole-Cell Extracts

Cells were washed 3 times with ice-cold phosphate-buffered saline (PBS, pH 7.4; Gibco) and lysed with ice-cold NP-40 lysis buffer (50 mM Tris-HCl, 150 mM NaCl, 1% NP-40, and 5 mM EDTA, pH 7.4 ± 0.2) with 1% proteinase inhibitor cocktail (Sigma-Aldrich, St. Louis, MO) and 1% Halt phosphatase inhibitor cocktail (Pierce, USA). Cells were incubated on ice for 30 minutes and centrifuged at 14,000 rpm at 4 °C for 10 minutes. The supernatant was collected, and protein concentration was measured by the bicinchoninic acid assay (BCA assay)[Pierce BCA Protein Assay Kit, MA, USA].

### Western Blot

Proteins were denatured by adding 6× Laemmli SDS sample buffer and heating for 5 minutes. SDS gel electrophoresis was performed with equivalent protein in each lane, followed by wet transfer of the protein to PVDF membrane. The membrane was blocked in TBS-T buffer (50 mmol/L Tris-HCl, pH 7.4, 150 mmol/L NaCl, and 0.05% Tween 20) containing 5% nonfat milk or BSA. The membrane was incubated overnight at 4 °C with primary antibody diluted in TBS-T containing 5% nonfat milk or BSA, followed by incubation for 1 hour at room temperature with the horseradish peroxidase–conjugated secondary antibody (Santa Cruz Biotechnology) diluted in TBS-T. The SuperSignal West Pico Chemiluminescent Substrate (Thermo Scientific, USA) was used for immunodetection.

### *In Vivo* Tumor Model

All animal work was conducted under protocols approved by the Mayo Clinic Institutional Animal Care and Use Committee. All procedures were performed according to the approved guidelines. For the pancreatic cancer orthotopic model, 8-week-old male SCID mice were obtained from the NCI Animal Production Program. Mice were anesthetized, and 1 × 10^6^ luciferase-labeled cells—control shRNA or NRP1 shRNA PANC-1 or BxPC-3 cells, resuspended in 100 μL of PBS—were injected into the pancreas of each mouse. For the lung cancer orthotopic model, the model was established in SCID mice as described before^52^. Briefly, animals were anesthetized using ketamine and placed in the left lateral decubitus position; 3 × 10^6^ luciferase-labeled cells—control shRNA or NRP1 shRNA A549 or H226 cells in PBS and 10 µL of Matrigel (BD Biosciences, USA)—were injected percutaneously into the right lung of the animals using 1-mL tuberculin syringes. The mice were returned to their cages after confirming they were fully recovered.

### Bioluminescent Imaging

Bioluminescent imaging of luciferase-expressing cells was detected in the orthotopic tumor models. Beetle Luciferin (Promega) (substrate for the luciferase-expressing tumor cells) was injected intraperitoneally at 150 mg/kg in PBS 15 minutes before imaging. Mice were anesthetized with 2% isoflurane and imaged once a week with a cooled CCD camera (IVIS system, Xenogen, USA). The exposure time was 1 second to 1 minute. Signal was displayed as photons/second/cm^2^/steradian and was quantified using the Living Image software (Caliper Life Sciences, USA) using the IVIS system 200 series (Xenogen Corp). At the end of the treatment, mice were sacrificed, and tumors were harvested for morphologic analysis and immunostaining.

### Immunohistochemical Staining

Tumors were removed and fixed in neutral buffered 10% formalin at room temperature for 24 hours before being embedded in paraffin and sectioned. Sections were deparaffinized, subjected to immunochemical staining, and imaged with an Olympus BX51 microscope.

### Statistical Analysis

Statistical analyses were performed with the SPSS 11.0 statistical software (SPSS, Inc). Data are presented as mean (SD). Statistical analysis was performed using 1-way analysis of variance, followed by SNK tests as post hoc testing. The Kruskal-Wallis test was used to evaluate for differences in categorical values, followed by Mann-Whitney U tests as post hoc testing. Analysis of NRP1 mRNA expression was performed using an unpaired *t* test with GraphPad software. Statistical significance was defined as *P* < 0.05, and a high level of statistical significance were defined as *P* < 0.01.

## Electronic supplementary material

Supplementary File

## References

[1] Eickholt BJ, Mackenzie SL, Graham A, Walsh FS, Doherty P (1999). Evidence for collapsin-1 functioning in the control of neural crest migration in both trunk and hindbrain regions. Development.

[2] Lampropoulou A, Ruhrberg C (2014). Neuropilin regulation of angiogenesis. Biochem Soc Trans.

[3] Graziani G, Lacal PM (2015). Neuropilin-1 as Therapeutic Target for Malignant Melanoma. Front Oncol.

[4] Prud’homme GJ, Glinka Y (2012). Neuropilins are multifunctional coreceptors involved in tumor initiation, growth, metastasis and immunity. Oncotarget.

[5] Kreuter M (2006). Correlation of neuropilin-1 overexpression to survival in acute myeloid leukemia. Leukemia.

[6] Meyerson HJ (2012). NRP-1/CD304 expression in acute leukemia: a potential marker for minimal residual disease detection in precursor B-cell acute lymphoblastic leukemia. Am J Clin Pathol.

[7] Sallam TH, El Telbany MA, Mahmoud HM, Iskander MA (2013). Significance of neuropilin-1 expression in acute myeloid leukemia. Turk J Haematol.

[8] Parikh AA (2003). Expression and regulation of the novel vascular endothelial growth factor receptor neuropilin-1 by epidermal growth factor in human pancreatic carcinoma. Cancer.

[9] Kawakami T (2002). Neuropilin 1 and neuropilin 2 co-expression is significantly correlated with increased vascularity and poor prognosis in nonsmall cell lung carcinoma. Cancer.

[10] Lantuejoul S (2003). Expression of VEGF, semaphorin SEMA3F, and their common receptors neuropilins NP1 and NP2 in preinvasive bronchial lesions, lung tumours, and cell lines. J Pathol.

[11] Hong TM (2007). Targeting neuropilin 1 as an antitumor strategy in lung cancer. Clin Cancer Res.

[12] Cao Y (2008). Neuropilin-1 upholds dedifferentiation and propagation phenotypes of renal cell carcinoma cells by activating Akt and sonic hedgehog axes. Cancer Res.

[13] Hu B (2007). Neuropilin-1 promotes human glioma progression through potentiating the activity of the HGF/SF autocrine pathway. Oncogene.

[14] Gagnon ML (2000). Identification of a natural soluble neuropilin-1 that binds vascular endothelial growth factor: *In vivo* expression and antitumor activity. Proc Natl Acad Sci USA.

[15] Miao HQ, Lee P, Lin H, Soker S, Klagsbrun M (2000). Neuropilin-1 expression by tumor cells promotes tumor angiogenesis and progression. FASEB J.

[16] Parikh AA (2004). Neuropilin-1 in human colon cancer: expression, regulation, and role in induction of angiogenesis. Am J Pathol.

[17] Gray MJ (2005). Neuropilin-1 suppresses tumorigenic properties in a human pancreatic adenocarcinoma cell line lacking neuropilin-1 coreceptors. Cancer Res.

[18] Cao Y (2012). VEGF exerts an angiogenesis-independent function in cancer cells to promote their malignant progression. Cancer Res.

[19] Marcus K (2005). Tumor cell-associated neuropilin-1 and vascular endothelial growth factor expression as determinants of tumor growth in neuroblastoma. Neuropathology.

[20] Xu J, Xia J (2013). NRP-1 silencing suppresses hepatocellular carcinoma cell growth *in vitro* and *in vivo*. Exp Ther Med.

[21] Yue B (2014). Knockdown of neuropilin-1 suppresses invasion, angiogenesis, and increases the chemosensitivity to doxorubicin in osteosarcoma cells - an *in vitro* study. Eur Rev Med Pharmacol Sci.

[22] Zeng F (2014). A monoclonal antibody targeting neuropilin-1 inhibits adhesion of MCF7 breast cancer cells to fibronectin by suppressing the FAK/p130cas signaling pathway. Anticancer Drugs.

[23] Adjei AA (2001). Ras signaling pathway proteins as therapeutic targets. Curr Pharm Des.

[24] Wennerberg K, Rossman KL, Der CJ (2005). The Ras superfamily at a glance. J Cell Sci.

[25] Lebrun JJ (2012). The Dual Role of TGFbeta in Human Cancer: From Tumor Suppression to Cancer Metastasis. ISRN Mol Biol.

[26] Cao Y (2010). Neuropilin-1 mediates divergent R-Smad signaling and the myofibroblast phenotype. J Biol Chem.

[27] Castro-Rivera E, Ran S, Brekken RA, Minna JD (2008). Semaphorin 3B inhibits the phosphatidylinositol 3-kinase/Akt pathway through neuropilin-1 in lung and breast cancer cells. Cancer Res.

[28] Barr MP (2005). A peptide corresponding to the neuropilin-1-binding site on VEGF(165) induces apoptosis of neuropilin-1-expressing breast tumour cells. Br J Cancer.

[29] Barr MP (2015). Vascular endothelial growth factor is an autocrine growth factor, signaling through neuropilin-1 in non-small cell lung cancer. Mol Cancer.

[30] Castro-Rivera E, Ran S, Thorpe P, Minna JD (2004). Semaphorin 3B (SEMA3B) induces apoptosis in lung and breast cancer, whereas VEGF165 antagonizes this effect. Proc Natl Acad Sci USA.

[31] Potiron VA (2007). Semaphorin SEMA3F affects multiple signaling pathways in lung cancer cells. Cancer Res.

[32] Tomizawa Y (2001). Inhibition of lung cancer cell growth and induction of apoptosis after reexpression of 3p21.3 candidate tumor suppressor gene SEMA3B. Proc Natl Acad Sci USA.

[33] Tse C, Xiang RH, Bracht T, Naylor SL (2002). Human Semaphorin 3B (SEMA3B) located at chromosome 3p21.3 suppresses tumor formation in an adenocarcinoma cell line. Cancer Res.

[34] Zhang G (2016). Neuropilin-1 (NRP-1)/GIPC1 pathway mediates glioma progression. Tumour Biol.

[35] Jarvis A (2010). Small molecule inhibitors of the neuropilin-1 vascular endothelial growth factor A (VEGF-A) interaction. J Med Chem.

[36] Jia H (2010). Neuropilin-1 antagonism in human carcinoma cells inhibits migration and enhances chemosensitivity. Br J Cancer.

[37] Wey JS (2005). Overexpression of neuropilin-1 promotes constitutive MAPK signalling and chemoresistance in pancreatic cancer cells. Br J Cancer.

[38] Downward J (2003). Targeting RAS signalling pathways in cancer therapy. Nat Rev Cancer.

[39] Katsuno Y, Lamouille S, Derynck R (2013). TGF-beta signaling and epithelial-mesenchymal transition in cancer progression. Curr Opin Oncol.

[40] Glinka Y, Stoilova S, Mohammed N, Prud’homme GJ (2011). Neuropilin-1 exerts co-receptor function for TGF-beta-1 on the membrane of cancer cells and enhances responses to both latent and active TGF-beta. Carcinogenesis.

[41] Grusch M (2010). The crosstalk of RAS with the TGF-beta family during carcinoma progression and its implications for targeted cancer therapy. Curr Cancer Drug Targets.

[42] Chow JY (2007). RAS/ERK modulates TGFbeta-regulated PTEN expression in human pancreatic adenocarcinoma cells. Carcinogenesis.

[43] Ji S (2015). ERK kinase phosphorylates and destabilizes the tumor suppressor FBW7 in pancreatic cancer. Cell Res.

[44] Zambirinis CP (2015). TLR9 ligation in pancreatic stellate cells promotes tumorigenesis. J Exp Med.

[45] Zhu F, Liu P, Li J, Zhang Y (2014). Eotaxin-1 promotes prostate cancer cell invasion via activation of the CCR3-ERK pathway and upregulation of MMP-3 expression. Oncol Rep.

[46] Koinuma D (2009). Chromatin immunoprecipitation on microarray analysis of Smad2/3 binding sites reveals roles of ETS1 and TFAP2A in transforming growth factor beta signaling. Mol Cell Biol.

[47] Louafi F, Martinez-Nunez RT, Sanchez-Elsner T (2010). MicroRNA-155 targets SMAD2 and modulates the response of macrophages to transforming growth factor-{beta}. J Biol Chem.

[48] El Ouaamari A (2016). SerpinB1 Promotes Pancreatic beta Cell Proliferation. Cell Metab.

[49] Longati P (2013). 3D pancreatic carcinoma spheroids induce a matrix-rich, chemoresistant phenotype offering a better model for drug testing. BMC Cancer.

[50] Hofmann I (2012). K-RAS mutant pancreatic tumors show higher sensitivity to MEK than to PI3K inhibition *in vivo*. PLoS One.

[51] Wang L, Zeng H, Wang P, Soker S, Mukhopadhyay D (2003). Neuropilin-1-mediated vascular permeability factor/vascular endothelial growth factor-dependent endothelial cell migration. J Biol Chem.

[52] Doki Y (1999). Mediastinal lymph node metastasis model by orthotopic intrapulmonary implantation of Lewis lung carcinoma cells in mice. Br J Cancer.

