# Supplementary material for: Genetic status of KRAS modulates the role of Neuropilin-1 in tumorigenesis
Source: Sci Rep. 2017 Oct 10;7:12877. doi: 10.1038/s41598-017-12992-2 (PMC5635066; doi:10.1038/s41598-017-12992-2)
Supplement: Supplementary file 1 — Supplementary File [file 41598_2017_12992_MOESM1_ESM.pdf]

Genetic status of *KRAS* modulates the role of Neuropilin-1 in tumorigenesis

Sneha Vivekanandhan[1, 2,$], Lijuan Yang[1,$], Ying Cao[1], Engfeng Wang[1,2]
Shamit K. Dutta[1,2], Anil K. Sharma[1,2], Debabrata Mukhopadhyay[1,2,*]

Department of Biochemistry and Molecular Biology, Mayo Clinic College of Medicine and Sciences, Rochester[1], MN and Jacksonville[2], FL. USA

[$] The first two authors contributed equally.

[*] Corresponding Author
Debabrata Mukhopadhyay, PhD, Department of Biochemistry and Molecular Biology, Mayo Clinic, 4500 San Pablo Rd Jacksonville, FL 32224
Email: mukhopadhyay.debabrata@mayo.edu

Supplementary Figure1

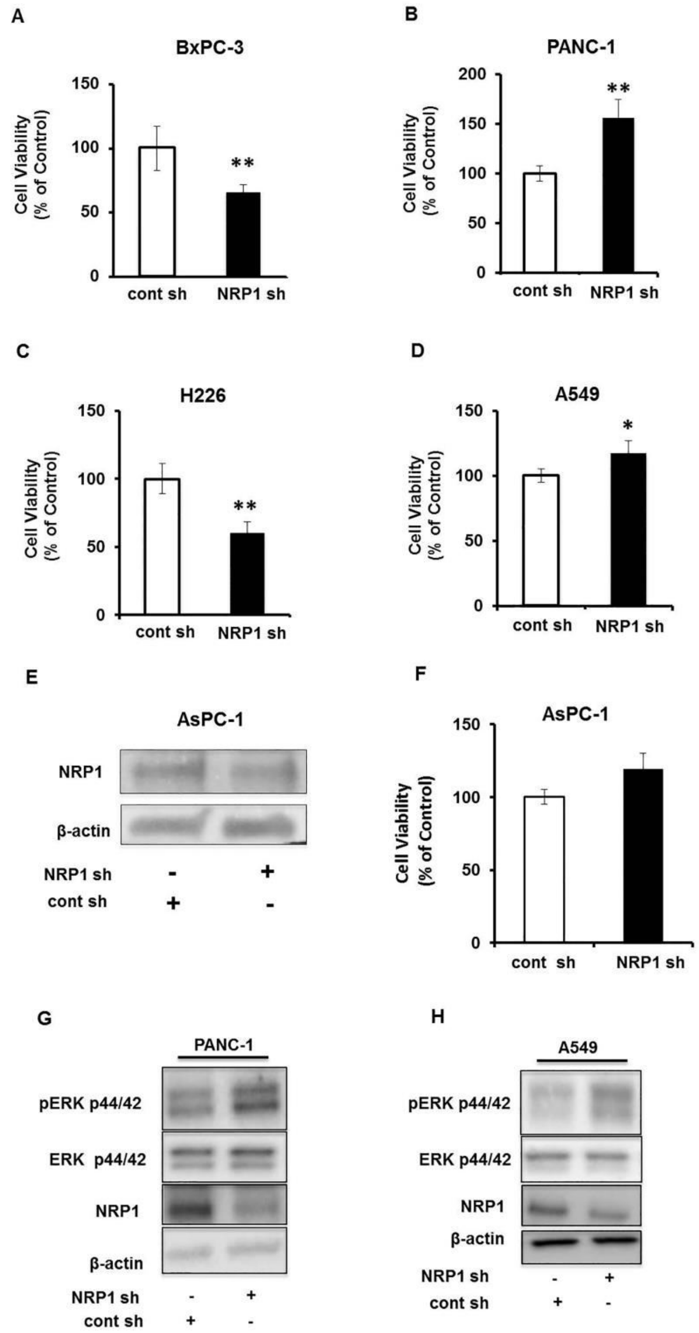

Supplementary Figure 2

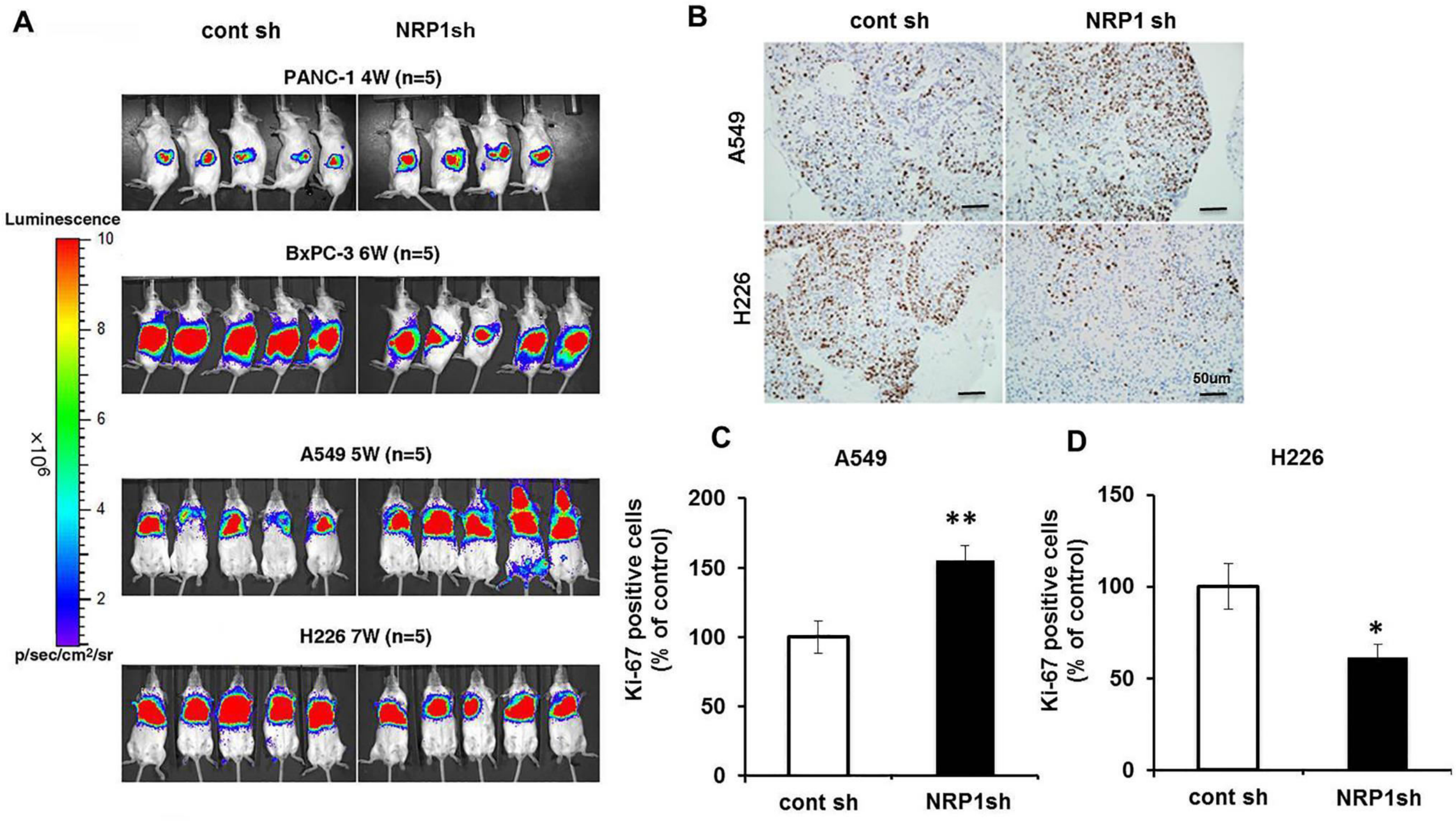

Supplementary Figure 3

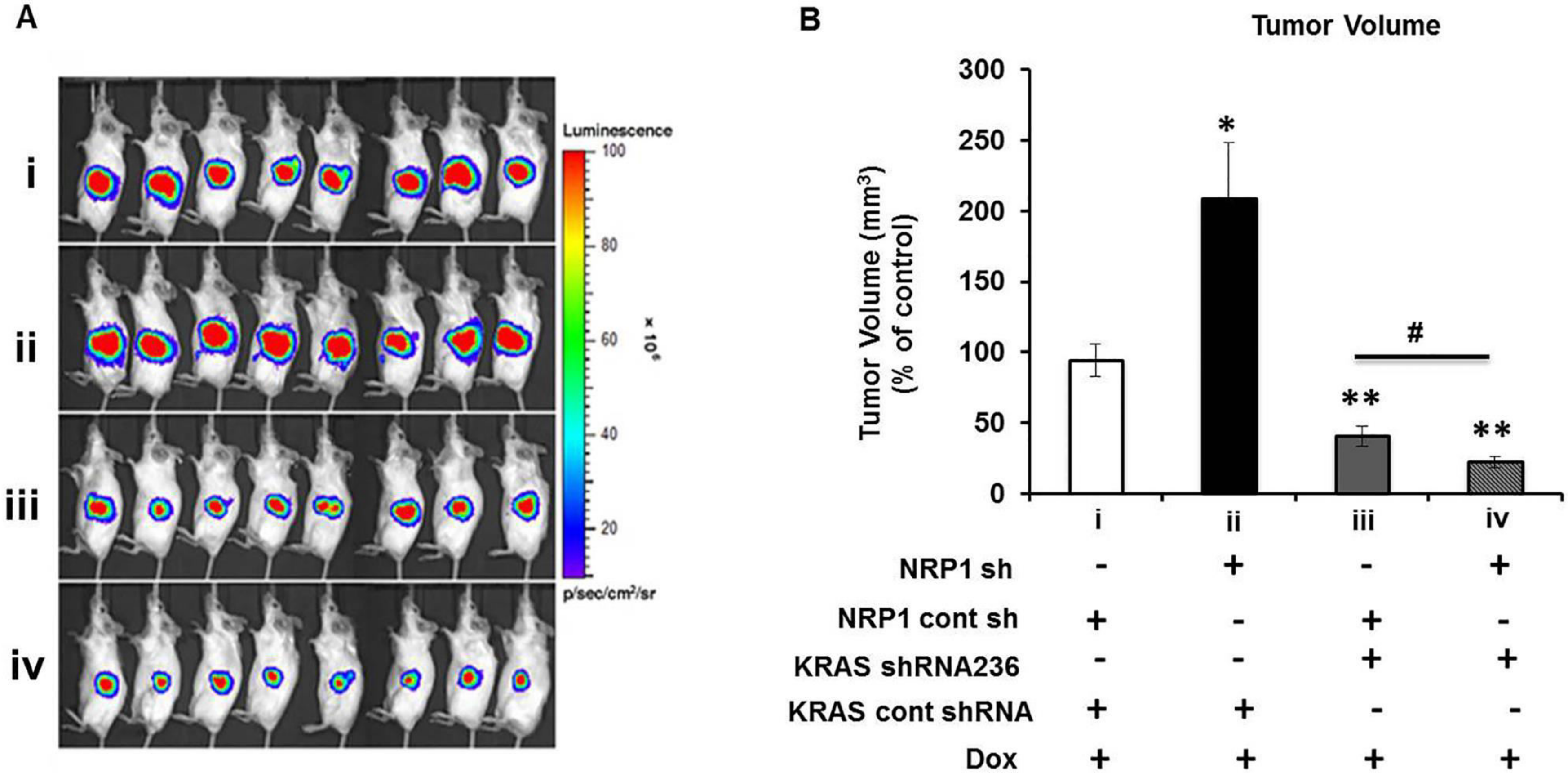

Supplementary Figure 4

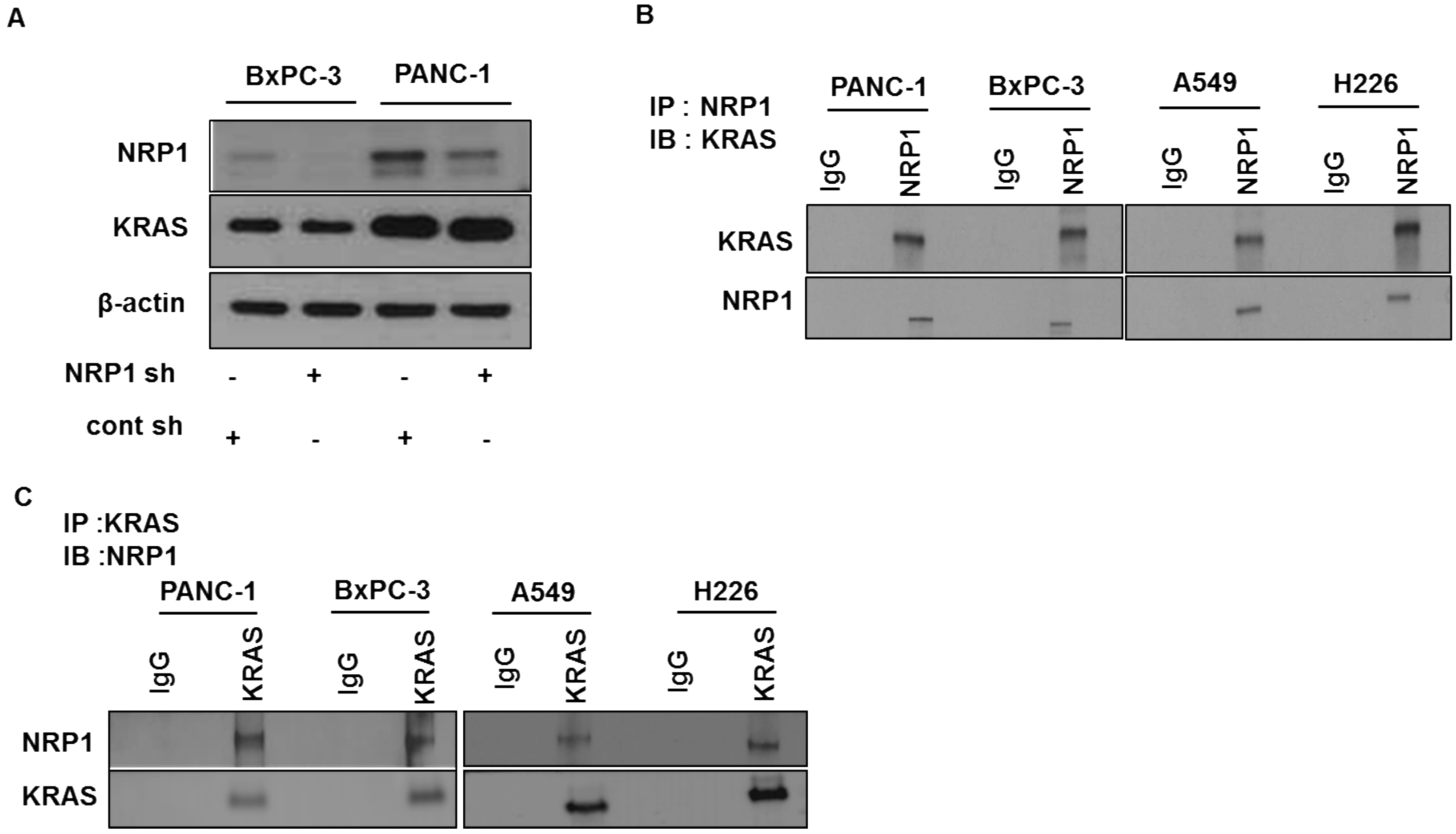

Supplementary Figure 5

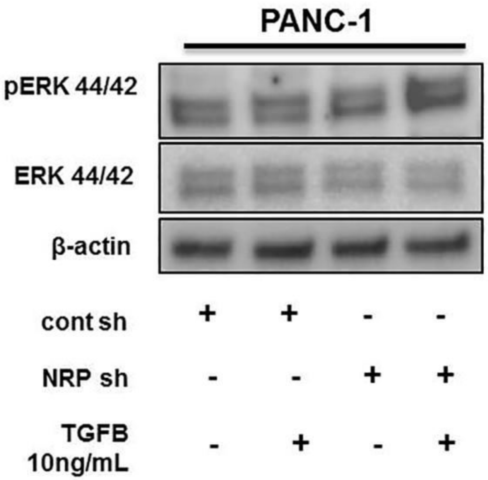

Supplementary Figure 6

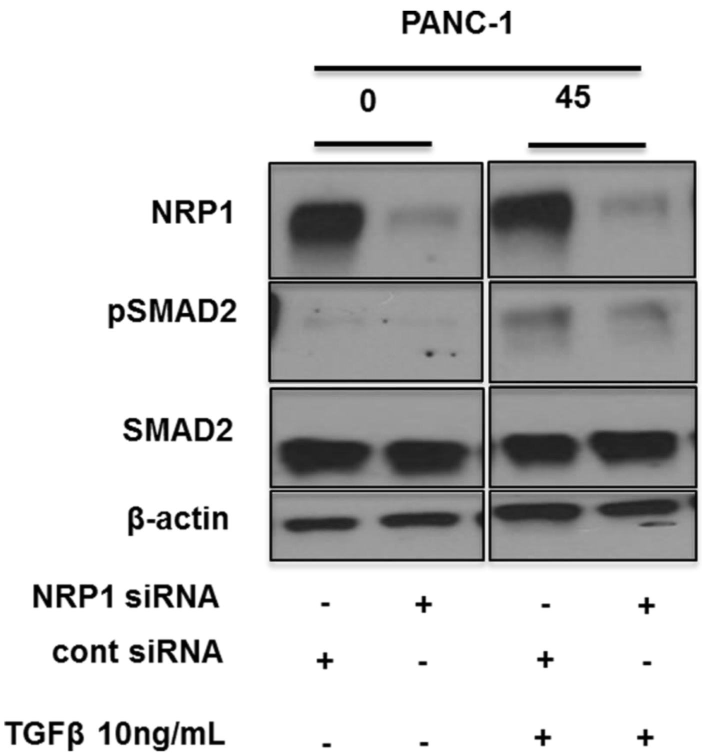

Supplementary Figure 7

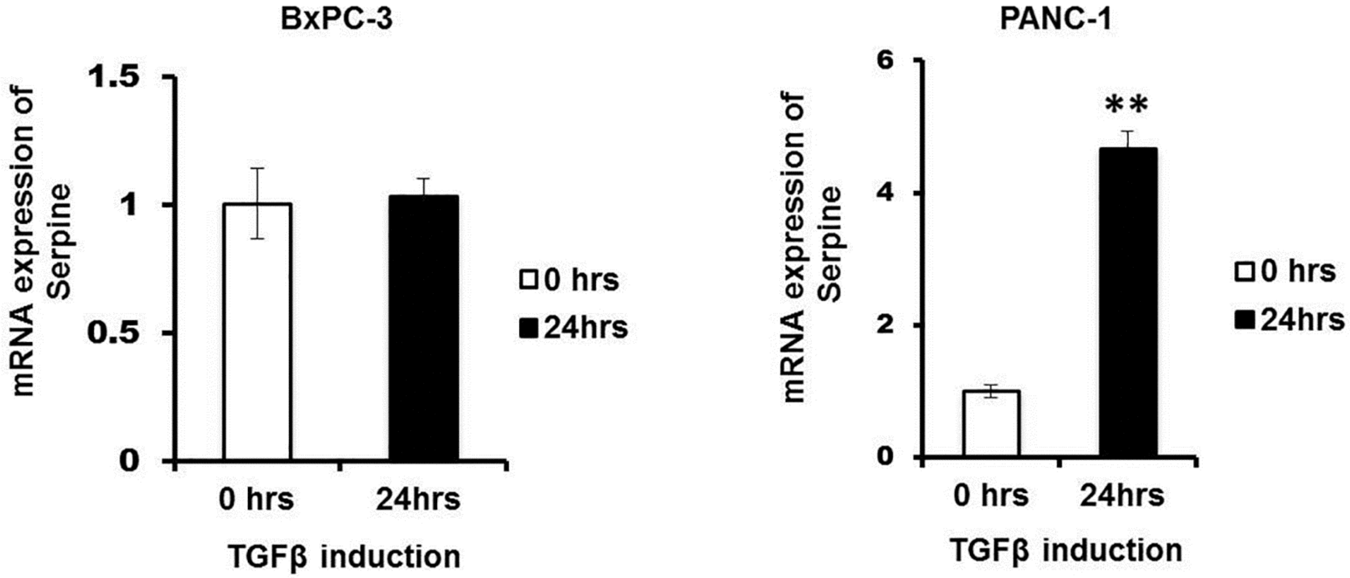

Supplementary Figure 8

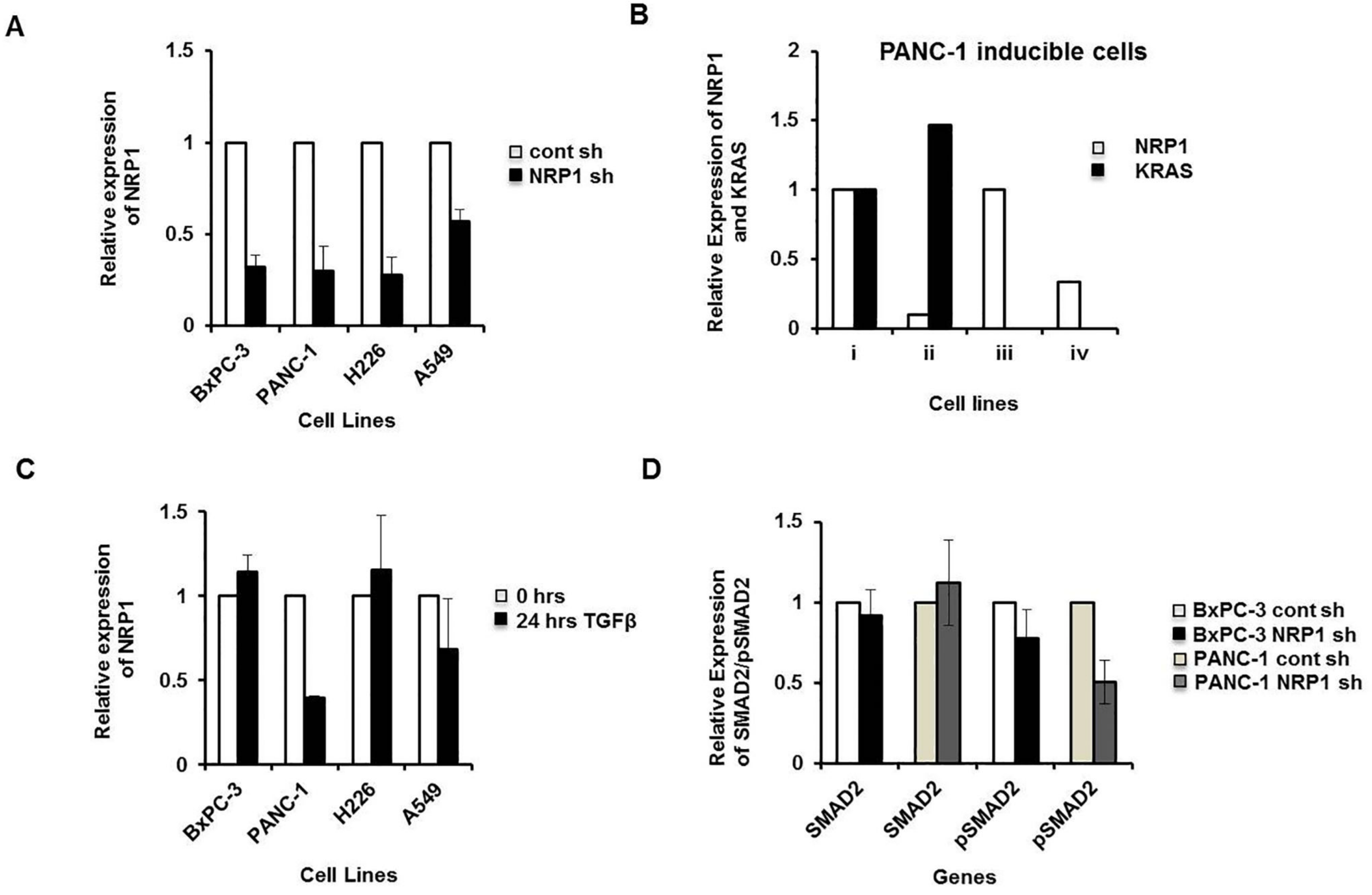

Supplementary Figures

S1: Oncogenic KRAS influences the role of NRP1 in tumorigenesis in pancreatic and lung cancers
S1 (A-D): Cell viability for different PDAC and NSCLC cell lines with NRP1 knockdown grown in 2D culture for 72 hours. Data are plotted as percentage of untreated control cells. A representative experiment out of three is shown. All data are presented as the mean ± SD of three independent experiments. Significant differences: *p < 0.05 *vs*. control group, **p < 0.01 *vs*. control group). **(E)** Western-blot analysis of NRP1 knockdown levels in PDAC cell line AsPC-1. **(F)** Cell viability for AsPC-1 with the reduced NRP1expression grown in 3D culture for 72 hours. **(G-H)** Western blot analysis of total and phosphorylated form of ERK p44/42 in PANC-1 and A549 cell lines with and without NRP1 knockdown.

S2: Role of NRP1 in tumorigenesis is different in wild-type vs mutant KRAS tumor models
S2 (A): Representative bioluminescent imaging of orthotopic SCID mice models implanted with different PDAC and NSCLC cells with NRP1 knockdown (n=5). **(B-D)** Immunohistochemistry staining for Ki-67 in different lung cancer cell lines with/out NRP1 knockdown and quantification of digital images for Ki-67 at 20X magnification. Significant differences: *p<0.05 *vs*. control group, **p<0.01 *vs*. control group.

S3: The role of NRP1 on tumorigenesis is oncogene KRAS dependent

S3 (A): Representative bioluminescent imaging of SCID mice orthotopically implanted with PANC-1 cells transfected**:** group (i) with NRP1 control- and KRAS control-shRNA; group (ii) with NRP1-shRNA and KRAS control-shRNA; group (iii) KRAS shRNA NRP1 control-shRNA and group (iv) KRAS shRNA and NRP1shRNA. **(B)** Comparison of tumor volume for the isolated tumors, representative data is shown for 8 animals in each group.

* *P*<.05 vs control; ** *P*<.01 vs control. # *P*< 05; ## *P*<. 01 between groups (iii) and (iv).

S4: NRP1 and KRAS are present in the same immunocomplex

S4 (A) Western blot showing levels of KRAS in PDAC cell lines with and without NRP1 reduced expression. **(B-C)** Coimmunoprecipitation assays showing that NRP1 and KRAS are present in the same immunocomplex.

S5: TGFβ induction increases phosphorylation of ERK44/42 in PANC-1 cells with reduced NRP1 expression

Western blot analysis showing levels of phosphorylated and total ERK in PANC-1 cells with and without NRP1 knockdown post 24hrs TGFβ treatment.

S6: TGFβ stimulation decreases phosphorylation of SMAD2 in PANC-1 cells with reduced NRP1 expression
Western blot showing levels of total and phosphoSMAD2 in PANC-1 cells treated with control or NRP1 siRNA 45mins after TGFβ stimulation.

S7: TGFβ induction increases mRNA expression levels of Serpine in PANC-1 cells
Real time quantifications of Serpine expression in pancreatic cancer cell lines with and without TGFβ induction for 24hrs. *$p<0.05$ *vs*. control group, **$p<0.01$ *vs*. control group.

S8: Quantifications of the western blots used
(A) Quantification for the western blots showing the reduced expression of NRP1 in PDAC and NSCLC cells. (B) Densitometry analysis for the western blots showing levels of NRP1 and KRAS in doxycycline inducible PANC-1 cells. (C) Quantification for the western blots showing the expression of NRP1 in PDAC and NSCLC cells post 24hrsTGFβ treatment. (D) Quantification for the western blots showing levels of total and phosphorylated SMAD2 in PDAC cell lines with and without reduced NRP1 expression.

Supplemental table 1: *KRAS* mutation status of associated cell lines and effect of NRP1 silencing on tumorigenesis

Tumor type	Cell type	KRAS mutation status	Result
Pancreatic cancer	PANC-1	MT	Reduced NRP1 expression promotes tumorigenesis
	AsPC-1	MT	Reduced NRP1 expression promotes tumorigenesis
	BxPC-3	WT	Reduced NRP1 expression inhibits tumorigenesis
Lung cancer	A549	MT	Reduced NRP1 expression promotes tumorigenesis
	H226	WT	Reduced NRP1 expression inhibits tumorigenesis

Supplemental table 2: The role of NRP1 in tumorigenesis in different cancers

Tumor type	Cell type	KRAS	Results	Ref
Neuroblastoma	SK-N-DZ（DZ）<br>SK-N-AS (AS)	WT	Overexpression promotes tumorigenesis	19
Glioma	U87MG	WT	Overexpression of NRP1 promotes tumor growth	53
Non-small cell lung cancer	CL1-0; CL1-1, CL1-5, CL1-5-F4	WT	Blockade of NRP1 suppresses tumor growth	11
Pancreatic cancer	PANC-1	MT	Reduced NRP1 expression promotes tumorigenesis	17
Prostate cancer	AT2.1	WT	Overexpression promotes tumorigenesis	15
Breast cancer	MCF-7	WT	Inhibition of NRP1 reduces cellular proliferation	22
Osteosarcoma	MG-63	WT	Overexpression promotes tumorigenesis	21
Hepatocellular carcinoma	HCC-LM6	WT	Reduced NRP1 expression inhibits tumorigenesis	20
Renal cancer	786-O<br>A498	WT	Reduced NRP1 expression inhibits tumorigenesis	12
Colon cancer	KM12	WT	Overexpression promotes tumorigenesis	16

Supplemental table 3: The sequences of siRNA and shRNA used in the study

siRNA/shRNA	Target sequences
NRP1 siRNA	5’-ACGGTCATAGACAGCACCATA-3’
NRP1 shRNA	5’-CCCTGTTGGTTTCATTTGAATA-3’
SMAD1 shRNA	5’-GCCGATGGACACAAACATGAT-3’
SMAD2 shRNA	5’-TGCTGTTGACAGTGAGCGAACTTG AGGTCTCATCAATTAATAGTGAAGCCA CAGATGTATTAATTGATGAGACCTCAAG TGTGCCTACTGCCTCGGA-3’
SMAD3 shRNA	5’-CTGTGTGAGTTCGCCTTCAAT-3’
SMAD5 shRNA	5’-GCCTAAACATTGGTGTTCAAT-3’
KRAS sh236:	5′-GATACAGCTAATTCAGAATC-3′
KRAS sh562:	5′-AGGCTCAGGACTTAGCAAGA-3′;
KRAS cont	5’-GGATAATGGTGATTGAGATGG-3′.
